# Type of intracranial hemorrhage after endovascular stroke treatment: association with functional outcome

**DOI:** 10.1136/jnis-2022-019474

**Published:** 2022-10-19

**Authors:** Wouter van der Steen, Nadinda A M van der Ende, Sven P R Luijten, Leon A Rinkel, Katinka R van Kranendonk, Henk van Voorst, Stefan D Roosendaal, Ludo F M Beenen, Jonathan M Coutinho, Bart J Emmer, Robert J van Oostenbrugge, Charles B L.M Majoie, Hester F Lingsma, Aad van der Lugt, Diederik W J Dippel, Bob Roozenbeek, MR CLEAN-NO IV, MR CLEAN-MED, and CONTRAST investigators

**Affiliations:** 1 Department of Neurology, Erasmus Medical Center, Rotterdam, The Netherlands; 2 Department of Radiology and Nuclear Medicine, Erasmus Medical Center, Rotterdam, The Netherlands; 3 Department of Neurology, Amsterdam UMC Locatie AMC, Amsterdam, The Netherlands; 4 Department of Radiology and Nuclear Medicine, Amsterdam UMC Locatie AMC, Amsterdam, The Netherlands; 5 Department of Biomedical Engineering and Physics, Amsterdam UMC Locatie AMC, Amsterdam, The Netherlands; 6 Department of Neurology, Maastricht University Medical Centre+, Maastricht, The Netherlands; 7 Department of Public Health, Erasmus Medical Center, Rotterdam, The Netherlands

**Keywords:** Stroke, Complication, Hemorrhage, Thrombectomy

## Abstract

**Background:**

Intracranial hemorrhage (ICH) is a frequent complication after endovascular stroke treatment.

**Objective:**

To assess the association of the occurrence and type of ICH after endovascular treatment (EVT) with functional outcome.

**Methods:**

We analyzed data from the MR CLEAN-NO IV and MR CLEAN-MED trials. Both trials included adult patients with ischemic stroke with a large vessel occlusion in the anterior circulation, who were eligible for EVT. ICH was classified (1) as asymptomatic or symptomatic (concomitant neurological deterioration of ≥4 points on the NIHSS, or ≥2 points on 1 NIHSS item), and (2) according to the Heidelberg Bleeding Classification. We used multivariable ordinal logistic regression analyses to assess the association of the occurrence and type of ICH with the modified Rankin Scale score at 90 days.

**Results:**

Of 1017 included patients, 331 (33%) had an asymptomatic ICH, and 90 (9%) had a symptomatic ICH. Compared with no ICH, both asymptomatic (adjusted common OR (acOR)=0.76; 95% CI 0.58 to 0.98) and symptomatic (acOR=0.07; 95% CI 0.04 to 0.14) ICH were associated with worse functional outcome. In particular, isolated parenchymal hematoma type 2 (acOR=0.37; 95% CI 0.14 to 0.95), combined parenchymal hematoma with hemorrhage outside infarcted brain tissue (acOR=0.17; 95% CI 0.10 to 0.30), and combined hemorrhages outside infarcted brain tissue (acOR=0.14; 95% CI 0.03 to 0.74) were associated with worse functional outcome than no ICH.

Strength of the association of ICH with functional outcome depends on the type of ICH. Although the association is stronger for symptomatic ICH, asymptomatic ICH after EVT is also associated with worse functional outcome.

**WHAT IS ALREADY KNOWN ON THIS TOPIC**

- Symptomatic intracranial hemorrhage after endovascular stroke treatment is strongly associated with poor outcome. However, the association of asymptomatic intracranial hemorrhage with functional outcome is not established. In addition, the association with functional outcome of subtypes of intracranial hemorrhage based on their anatomic description is incompletely understood.

**WHAT THIS STUDY ADDS**

- This study provides a better understanding of the associations of the occurrence and subtypes of intracranial hemorrhage after endovascular stroke treatment with functional outcome. Moreover, we have now established that asymptomatic intracranial hemorrhage is also associated with worse functional outcome.

**HOW THIS STUDY MIGHT AFFECT RESEARCH, PRACTICE OR POLICY**

- The results of this study can be used to better assess the clinical significance of each intracranial hemorrhage occurring after endovascular stroke treatment. In addition, our study supports the notion that the distinction between symptomatic and asymptomatic intracranial hemorrhage should be refined.

## Introduction

Intracranial hemorrhage (ICH) is a frequent complication after endovascular treatment (EVT) for acute ischemic stroke.^1^ According to the Heidelberg Bleeding Classification, an ICH is classified as symptomatic when it is associated with concomitant neurological deterioration (ie, an increase of ≥4 points on the National Institutes of Health Stroke Scale (NIHSS) or ≥2 points on a specific NIHSS item).^2^ In addition, the Heidelberg Bleeding Classification can be used to classify ICH based on imaging characteristics and anatomic description: hemorrhagic transformation of infarcted brain tissue is classified as hemorrhagic infarction type 1, hemorrhagic infarction type 2, or parenchymal hematoma type 1; hemorrhage within and beyond infarcted brain tissue is classified as parenchymal hematoma type 2; and hemorrhages outside infarcted brain tissue are classified as subarachnoid hemorrhage, subdural hematoma, intraventricular hemorrhage, or remote parenchymal hematoma.

Symptomatic ICH after reperfusion therapy is strongly associated with poor outcome.^3 4^ However, the association of asymptomatic ICH with functional outcome is not established.^5–7^ Moreover, the association with functional outcome for subtypes of ICH based on anatomic description is not always clear. A better understanding of these potential associations would provide more insight into the clinical significance of each type of ICH occurring after EVT.

The aim of this study was to assess the association of the occurrence, symptomatic status, and type of ICH after EVT with functional outcome.

## Methods

### Study design and patients

This is a post hoc observational study with data from the Multicenter Randomized CLinical trial of Endovascular treatment for Acute ischemic stroke in the Netherlands (MR CLEAN)-NOIV and MR CLEAN-MED.^8 9^ Both studies were phase III multicenter clinical trials with randomized group assignment, open label treatment, and blinded outcome evaluation. In MR CLEAN-NO IV, EVT plus intravenous thrombolytics was compared with EVT alone. In MR CLEAN-MED, EVT with routine periprocedural use of intravenous antithrombotics (ie, aspirin or unfractionated heparin) was compared with EVT without routine periprocedural use of antithrombotics. Both studies started inclusion in January 2018, and included adult patients with a large vessel occlusion in the anterior circulation eligible for EVT. MR CLEAN-NO IV included patients who presented directly to a hospital that was capable of providing EVT, and who were eligible for intravenous thrombolytics ≤4.5 hours after stroke onset or last seen well. Inclusion was completed in October 2020. MR CLEAN-MED included patients who were eligible for EVT ≤6 hours after stroke onset or last seen well. In January 2021, inclusion in MR CLEAN-MED was stopped owing to safety concerns about the study treatments. The studies ran in parallel to each other in intervention centers in the Netherlands, and MR CLEAN-NO IV also included patients in Belgium and France. Both studies used a deferred consent procedure in accordance with national legislation.^10^ For the current analysis, we selected patients with deferred consent for 3-month clinical follow-up, who were treated with EVT (defined as entry into the angiography suite and receiving arterial puncture), and had available follow-up imaging of sufficient quality to assess the occurrence of ICH.

Protocols and results of both MR CLEAN-NO IV and MR CLEAN-MED have been published previously.^8 9 11 12^ Both protocols were approved by a central medical ethics committee. De-identified data collected for the studies will be made available to others on reasonable request. Data can be requested with a proposal at the website of the CONTRAST consortium (www.contrast-consortium.nl), or by sending an e-mail to the corresponding author.

### Outcomes

In both MR CLEAN-NO IV and MR CLEAN-MED, all patients were followed up until final assessment at 90 days. Clinical outcome data at 90 days were collected centrally through standardized telephone interviews by trained research nurses. A blinded outcome committee adjudicated the primary outcome (modified Rankin Scale (mRS) score) data based on the interview reports. In both trials, standard follow-up imaging (CT or MRI) was performed at 24 hours after EVT and at 5–7 days after EVT (or earlier at discharge). In addition, the treating physician could decide to perform imaging based on local protocols (eg, in cases of neurological deterioration). An imaging core committee consisting of neuroradiologists and interventionalists, masked to all clinical data except to the side of stroke, assessed the images. Among others, they assessed ICH occurrence and classified each ICH according to the Heidelberg Bleeding Classification. A blinded serious adverse event committee assessed whether an ICH was asymptomatic or symptomatic, also based on the Heidelberg Bleeding Classification (ie, concomitant increase of ≥4 points on the NIHSS or ≥2 points on a specific NIHSS item).

### Statistical analysis

We formatted descriptive tables stratified for no ICH occurrence, asymptomatic ICH occurrence, or symptomatic ICH occurrence. We performed univariable and multivariable proportional odds regression analyses to assess the effect of the occurrence and type of ICH on the mRS score at 90 days. We analyzed the association of any ICH compared with no ICH (model 1); the association of ICH, classified as asymptomatic and symptomatic, compared with no ICH (model 2); and the association of each type of ICH classified according to the Heidelberg Bleeding Classification compared with no ICH (model 3). In model 3, patients with an isolated subdural hematoma, isolated intraventricular hemorrhage, or isolated remote parenchymal hematoma were merged into one subgroup, because of the small number of patients in these categories. In addition, patients with a combination of two or more hemorrhage types were analyzed in separate groups (ie, combined hemorrhagic infarction with hemorrhage outside infarcted brain tissue, combined parenchymal hematoma with hemorrhage outside infarcted brain tissue, or combined hemorrhages outside infarcted brain tissue). The effect estimates of all multivariable models were adjusted for age, sex, pre-stroke mRS score, baseline systolic blood pressure, history of diabetes mellitus, history of myocardial infarction, prior use of antithrombotics, baseline NIHSS score, stroke onset to groin puncture time, treatment with intravenous thrombolytics, post-EVT extended treatment in cerebral infarction score, final infarct volume, periprocedural treatment with aspirin, and periprocedural treatment with unfractionated heparin.

All statistical analyses were performed using R version 4.1.1. (www.cran.r-project.org) with the packages Hmisc, rms, and tableone. For univariable and multivariable regression analyses, we replaced missing independent variables with multiple imputation using the aregImpute function. We generated five multiple imputation sets, in which we used three knots for continuous variables.

## Results

### Patients

Of 547 patients randomized in MR CLEAN-NO IV, eight patients had not given consent for 3-month clinical follow-up, and of 663 patients randomized in MR CLEAN-MED, 35 patients had not given consent for 3-month clinical follow-up (online supplemental figure I). Of the remaining patients, we excluded 69 patients of MR CLEAN-NO IV and 81 patients of MR CLEAN-MED who had not been treated with EVT (n=36), or had no follow-up imaging with sufficient quality to assess ICH occurrence (n=114). In total, 1017 patients were available for the analysis.

10.1136/jnis-2022-019474.supp1 Supplementary data

### Patient characteristics

Median age of included patients was 72 (IQR 63–80) years, 562 (55%) were men, and median baseline NIHSS score was 16 (IQR 9–20) (table 1). Most patients had an M1 occlusion (557 (55%)), followed by an internal carotid artery occlusion (252 (25%)), and an M2 occlusion (201 (20%)). In four (0.4%) patients the imaging core committee found no occlusion on baseline imaging. In total, 642 (63%) patients received intravenous thrombolytics, 265 (26%) periprocedural intravenous aspirin, and 366 (36%) periprocedural intravenous unfractionated heparin. Of the included patients, 620 (61%) only had CT as follow-up imaging, 327 (32%) only MRI, and 70 (6.9%) both CT and MRI. In total, 182/14 238 (1.3%) data points of independent variables used for the regression analyses were missing and imputed.

**Table 1:** Baseline characteristics of the study population, stratified by ICH occurrence and concomitant neurological deterioration

Characteristics	No ICH (n=596)	Asymptomatic ICH (n=331)	Symptomatic ICH (n=90)	Missing
Age, years; median (IQR)	71 (62–79)	72 (63–80)	75 (66–83)	0
Men, n (%)	325 (55)	193 (58)	44 (49)	0
Trial, n (%)				0
MR CLEAN-NO IV	300 (50)	145 (44)	25 (28)	
MR CLEAN-MED	296 (50)	186 (56)	65 (72)	
Pre-stroke mRS score, n (%)				2
0	419 (70)	230 (70)	51 (57)	
1	107 (18)	56 (17)	22 (24)	
2	54 (9.1)	33 (10)	10 (11)	
≥3	15 (3.0)	11 (3.3)	7 (7.8)	
Medical history				
Atrial fibrillation, n (%)	101 (17)	63 (19)	21 (23)	0
Diabetes mellitus, n (%)	80 (13)	59 (18)	19 (21)	0
Myocardial infarction, n (%)	72 (12)	29 (8.8)	13 (14)	0
Prior antithrombotic drug use				
Antiplatelet, n (%)	184 (31)	109 (33)	41 (46)	0
Coumarine, n (%)"	40 (6.7)	23 (6.9)	10 (11)	0
Direct oral anticoagulant, n (%)	36 (6.0)	21 (6.3)	6 (6.7)	0
Heparin, n (%)	4 (0.7)	1 (0.3)	1 (1.1)	0
SBP at baseline, mm Hg; mean (SD)	150 (25)	151 (25)	156 (24)	7
NIHSS score at baseline; median (IQR)	15 (8–19)	16 (11–20)	17 (10–21)	18
ASPECTS at baseline NCCT; median (IQR)	9 (8–10)	9 (7–10)	9 (8–10)	3
Level of occlusion on CTA, n (%)				3
ICA or ICA-T	148 (25)	84 (25)	20 (22)	
M1	331 (56)	183 (55)	43 (48)	
M2	112 (19)	63 (19)	26 (29)	
None	2 (0.3)	1 (0.3)	1 (1.1)	
Treatment with intravenous thrombolytics, n (%)	374 (63)	204 (62)	64 (71)	0
Onset to groin puncture time, median (IQR)	150 (115–195)	161 (130–218)	194 (145–254)	20
Periprocedural treatment with aspirin, n (%)	128 (21)	91 (27)	46 (51)	1
Periprocedural treatment with heparin, n (%)	193 (32)	126 (38)	47 (52)	2
Post-EVT eTICI score, n (%)				56
0	46 (8.3)	26 (8.2)	6 (6.7)	
1	11 (2.0)	5 (1.6)	0 (0.0)	
2a	43 (7.8)	32 (10)	9 (10)	
2b	107 (19)	85 (27)	22 (24)	
2c	71 (13)	39 (12)	11 (12)	
3	275 (50)	132 (41)	41 (46)	

Continuous variables are presented as median and IQR or mean and SD. Categorical variables are presented as frequencies (n) and percentages (%).

ASPECTS, Alberta Stroke Program Early CT Score; CTA, CT angiography; eTICI, extended thrombolysis in cerebral infarction; EVT, endovascular treatment; ICA(-T), internal carotid artery (terminus); ICH, intracranial hemorrhage; mRS, modified Rankin Scale; mRS, modified Rankin Scale; NCCT, non-contrast CT; NIHSS, National Institutes of Health Stroke Scale; SBP, systolic blood pressure.

### Outcomes

**Figure 1:**
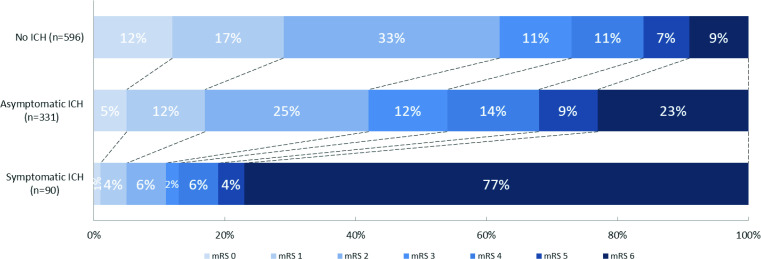
Distribution of modified Rankin Scale (mRS) scores at 90 days for patients with no intracranial hemorrhage (ICH), asymptomatic ICH, and symptomatic ICH. There was a significant shift towards worse functional outcomes for both patients with asymptomatic ICH versus patients with no ICH (adjusted common odds ratio (acOR)=0.76; 95% CI 0.58 to 0.98), and for patients with symptomatic ICH versus patients with no ICH (acOR=0.07; 95% CI 0.04 to 0.14).

**Figure 2:**
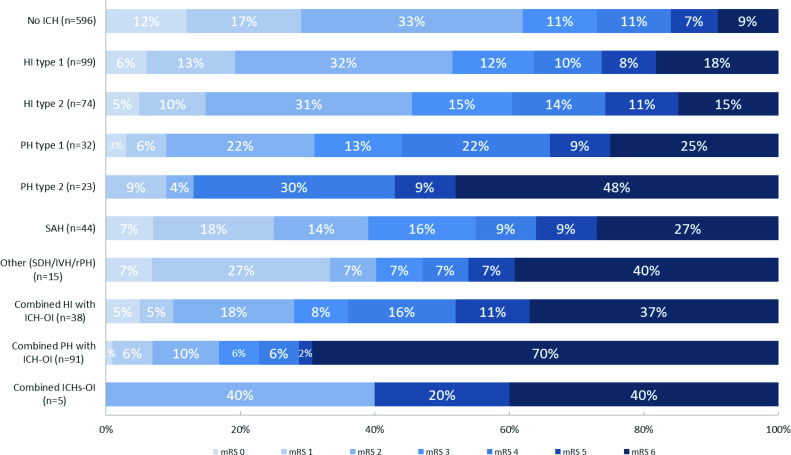
Distribution of modified Rankin Scale (mRS) scores at 90 days for patients with ICH stratified for imaging characteristics and anatomic description. HI, hemorrhagic Infarction; ICH, intracranial hemorrhage; ICH-OI, intracranial hemorrhage outside infarcted brain tissue; IVH, intraventricular hemorrhage; PH, parenchymal hematoma; SAH, subarachnoid hemorrhage; SDH, subdural hematoma; rPH, remote parenchymal hematoma.

Of 1017 included patients, 331 (33%) had an asymptomatic ICH, and 90 (9%) had a symptomatic ICH (online supplemental table I). Asymptomatic ICHs mainly included isolated hemorrhagic infarction type 1 (99 (30%)), isolated hemorrhagic infarction type 2 (74 (22%)), and isolated subarachnoid hemorrhage (41 (12%)). Symptomatic ICHs mainly included combined parenchymal hematoma with hemorrhage outside infarcted brain tissue (59 (66%)), and isolated parenchymal hematoma type 2 (11 (12%)). None of the isolated hemorrhagic infarctions type 1 or 2 were classified as symptomatic ICH. Number of hemorrhages per subclassification of hemorrhages classified as ‘Other’ and of combined hemorrhages are given in the supplements (online supplemental tables II–V). Patients with any ICH more often had poor functional outcome than patients with no ICH (online supplemental figure II). Distribution of mRS scores at 90 days for patients with no ICH, asymptomatic ICH, and symptomatic ICH are given in figure 1. Distribution of mRS scores at 90 days for patients with ICH, stratified for type of ICH are given in figure 2.

Compared with no ICH, the occurrence of any ICH was associated with worse functional outcome in both univariable (common OR (cOR)=0.31; 95% CI 0.25 to 0.39) and multivariable (adjusted cOR=0.59; 95% CI 0.46 to 0.76) regression analyses (table 2). Subdivided by concomitant neurologic deterioration, both asymptomatic (acOR=0.76; 95% CI 0.58 to 0.98) and symptomatic ICH (acOR=0.07; 95% CI 0.04 to 0.14) were associated with worse functional outcome than no ICH. Classified by type of ICH, all estimates pointed towards worse functional outcome, but only isolated parenchymal hematoma type 2 (acOR=0.37; 95% CI 0.14 to 0.95), combined parenchymal hematoma with hemorrhage outside infarcted brain tissue (acOR=0.17; 95% CI 0.10 to 0.30), and combined hemorrhages outside infarcted brain tissue (acOR=0.14; 95% CI 0.03 to 0.74) were significantly associated with a worse functional outcome than no ICH occurrence.

**Table 2:** Regression analyses of the association of any intracranial hemorrhage (model 1), intracranial hemorrhage classified according to symptoms (model 2), and intracranial hemorrhage classified according to the Heidelberg Bleeding Classification (model 3) with the modified Rankin Scale score at 90 days

	Common OR (95% CI)	Adjusted common odds ratio (95% CI)*
*Model 1*		
Any intracranial hemorrhage	0.31 (0.25 to 0.39)	0.59 (0.46 to 0.76)
*Model 2*		
Asymptomatic intracranial hemorrhage	0.44 (0.35 to 0.57)	0.76 (0.58 to 0.98)
Symptomatic intracranial hemorrhage	0.04 (0.02 to 0.07)	0.07 (0.04 to 0.14)
*Model 3*		
Isolated hemorrhagic infarction type 1	0.61 (0.42 to 0.89)	0.76 (0.51 to 1.13)
Isolated hemorrhagic infarction type 2	0.54 (0.36 to 0.82)	1.00 (0.64 to 1.56)
Isolated parenchymal hematoma type 1	0.32 (0.17 to 0.59)	0.67 (0.35 to 1.30)
Isolated parenchymal hematoma type 2	0.12 (0.06 to 0.27)	0.37 (0.14 to 0.95)
Isolated subarachnoid hemorrhage	0.44 (0.25 to 0.77)	0.55 (0.30 to 1.03)
Other isolated hemorrhage (SDH/IVH/rPH)	0.35 (0.12 to 1.01)	0.50 (0.17 to 1.49)
Combined hemorrhagic infarction with hemorrhage outside infarcted brain tissue	0.23 (0.13 to 0.42)	0.52 (0.27 to 1.03)
Combined parenchymal hematoma with hemorrhage outside infarcted brain tissue	0.06 (0.04 to 0.10)	0.17 (0.10 to 0.30)
Combined hemorrhages outside infarcted brain tissue	0.20 (0.04 to 1.02)	0.14 (0.03 to 0.74)

Effect estimates are presented as common odds ratios (OR) and adjusted common odds ratios (aOR) with 95% confidence intervals (CI). The reference for all models was ‘no intracranial hemorrhage’.

*Adjusted for age, sex, pre-stroke mRS score, history of hypertension, history of diabetes mellitus, history of myocardial infarction, prior use of antithrombotics, baseline NIHSS score, door intervention center to groin puncture time, intravenous thrombolytics, post-EVT eTICI score, final infarct volume, periprocedural treatment with aspirin, and periprocedural treatment with unfractionated heparin.

eTICI, extended thrombolysis in cerebral infarction; EVT, endovascular treatment; IVH, intraventricular hemorrhage; mRS, modified Rankin Scale; NIHSS, National Institutes of Health Stroke Scale; rPH, remote parenchymal hematoma; SDH, subdural hematoma.

## Discussion

In this post hoc study with combined data of two randomized controlled trials, we found that in patients with a stroke treated with EVT both asymptomatic and symptomatic ICH were associated with worse functional outcome. Strength of the association was stronger for symptomatic ICH and was dependent on the type of ICH, based on imaging characteristics and anatomic description.

Any ICH has been associated with worse functional outcome before, but not all studies found a significant association.^4 6 13^ However, studies that did not find a significant association, did show a strong trend towards worse functional outcome. The results of our study indicate that the occurrence of any ICH indeed has a negative impact on functional outcome. Both asymptomatic and symptomatic ICH were associated with worse functional outcome in our study. For symptomatic ICH this is not surprising, as several other studies previously showed a strong association with worse functional outcome.^4 6 13 14^ However, for asymptomatic ICH, earlier results have been less clear. Although asymptomatic ICH has previously been associated with higher mortality and longer stay on the intensive care unit, earlier studies only showed a non-significant trend towards worse functional outcome.^6 15^ As the direction and effect of the different studies are consistent, we consider it likely that the significant association found in our study is correct. Apparently, an asymptomatic ICH is not asymptomatic after all.

As reaffirmed in our study, the strength of the association of an ICH depends on its type, based on imaging characteristics and anatomic description.^5 6^ When combing results of earlier studies and our study, mainly parenchymal hematomas, and combined hemorrhages seem to be associated with a worse functional outcome.^5 6 16 17^ This while isolated hemorrhagic infarctions appear to have no association with functional outcome. The impact of isolated hemorrhages outside infarcted brain tissue on outcome remains less clear. We did not find a significant association, but this might be caused by a lack of power due to the relatively small groups, even after combining isolated subdural hematomas, isolated intraventricular hemorrhages, and isolated remote parenchymal hematomas. Previous studies on the association of isolated hemorrhages outside infarcted brain tissue with functional outcome also had relatively small sample sizes.^4 6 18 19^ Further meta-analysis with individual patient data may be required to gain more clarity on this issue.

Interestingly, none of the hemorrhagic infarctions in our study was classified as symptomatic. In addition, the majority of isolated parenchymal hematoma type 1, isolated subarachnoid hemorrhage, and combined hemorrhagic infarction with hemorrhage outside infarcted brain tissue was asymptomatic, whereas the majority of combined parenchymal hematoma with hemorrhage outside infarcted brain tissue was symptomatic. The distribution of hemorrhage types in our analyses is comparable to earlier studies on this topic.^5 6 16^ However, we found slightly higher overall risks of intracranial hemorrhage and symptomatic intracranial hemorrhage than most other studies. This is probably caused by the increased risk of hemorrhage in the subgroup of patients allocated to periprocedural unfractionated heparin or aspirin in the MR CLEAN-MED trial.^20^

In the Heidelberg Bleeding Classification, an ICH is considered symptomatic when an increase of ≥4 points on the NIHSS or ≥2 points on a specific NIHSS item occurs.^2^ This limit was set because this was the limit at which a change in neurological status was considered to be potentially associated with a worsened long-term prognosis. However, as our study found that by using this definition asymptomatic ICHs are also associated with worse functional outcome, this definition might need refinement.

Several studies have investigated the determinants of both asymptomatic and symptomatic ICH after endovascular stroke treatment; however, robust evidence is limited.^21^ Now that the impact of these hemorrhages on functional outcome becomes more clear, it seems wise to put even more effort into trying to predict and, more importantly, prevent these hemorrhages. With this, it should be evaluated whether determinants differ according to location and anatomic description.^22 23^ First, because we have reaffirmed that their prognostic value differs, and second because their underlying mechanisms differ.^24^

Lastly, our results suggest that standard follow-up imaging even in patients without neurological deterioration may be of added value. The implementation of standard follow-up imaging may help clinicians in estimating the prognosis of the patient. In addition, it can play a key role in guiding care-related decisions like the antithrombotic treatment regimen.^25 26^ Whether it is best to use MRI or CT for this indication should be evaluated in other studies. On the one hand, CT could be preferred as it is faster, cheaper, and more widely available. On the other hand, MRI could be preferred as its sensitivity and inter-rater agreement is better.^27^

### Limitations

First, we used the data of two randomized controlled trials, potentially limiting the generalizability of the results. However, inclusion and exclusion criteria in the trials were lenient, and in the analyses we adjusted for the evaluated study treatments (ie, treatment with intravenous thrombolytics, periprocedural aspirin, or periprocedural unfractionated heparin). In addition, the study population is representative of clinical practice. Second, some patients in the MR CLEAN-NO IV and MR CLEAN-MED trial were excluded because they did not provide deferred consent for primary outcome assessment, potentially introducing a selection bias. However, in the main papers of the trials, sensitivity analyses on primary safety outcomes (ie, symptomatic ICH and death from any cause) including data of these patients showed comparable results to those in the main analyses. This indicates that there was no selective withdrawal of patients, limiting the risk of a bias. Third, follow-up CT scans were not always accompanied by a dual-energy scan to differentiate contrast staining from ICH. However, dual-energy scans change the radiological diagnosis of post-treatment ICH to ‘contrast staining only’ in only a small proportion of patients.^28^ Last, we used different follow-up imaging modalities (ie, non-contrast CT and MRI). MRI depicts more hemorrhages and has higher intraobserver and interobserver agreement than CT.^27^ This might have affected point estimates of the investigated associations. It would have been interesting to assess whether there was an interaction between follow-up imaging modality and effect of ICH on functional outcome. However, because patients with a poor neurological status more frequently underwent CT than MRI, these results would be confounded by indication.

## Conclusion

The strength of the association of ICH with functional outcome depends on the type of ICH determined by concomitant neurological deterioration or anatomic description. Although the association is stronger for symptomatic ICH, we have now established that asymptomatic ICH after EVT is also associated with worse functional outcome.

10.1136/jnis-2022-019474.supp2 Supplementary data

## Data Availability

Data are available upon reasonable request. De-identified data collected for the studies will be made available to others upon reasonable request. Data can be requested with a proposal at the website of the CONTRAST consortium (www.contrast-consortium.nl), or by sending an email to the corresponding author.
