# Supplementary material for: Type of intracranial hemorrhage after endovascular stroke treatment: association with functional outcome
Source: J Neurointerv Surg. 2022 Oct 19;15(10):971–6. doi: 10.1136/jnis-2022-019474 (PMC10511981; doi:10.1136/jnis-2022-019474)
Supplement: Supplementary data [file jnis-2022-019474supp001.pdf]

SUPPLEMENTARY MATERIAL ON:

TYPE OF INTRACRANIAL HEMORRHAGE AFTER ENDOVASCULAR STROKE TREATMENT: ASSOCIATION WITH FUNCTIONAL OUTCOME

Wouter van der Steen, MD[1,2]; Nadinda van der Ende, MD[1,2]; Sven Luijten, MD[2]; Leon A. Rinkel, MD[3]; Katinka R. van Kranendonk, MD[4]; Henk van Voorst, MD[4,5]; Stefan D. Roosendaal, MD, PhD[4]; Ludo F.M. Beenen, MD, PhD[4]; Jonathan M. Coutinho, MD, PhD[3]; Bart J. Emmer, MD, PhD[4]; Robert J. van Oostenbrugge, MD, PhD[6]; Charles B.L.M. Majoie, MD, PhD[4]; Hester F. Lingsma, PhD[7]; Aad van der Lugt, MD, PhD[2]; Diederik W.J. Dippel, MD, PhD[1]; Bob Roozenbeek, MD, PhD[1,2]; on behalf of the MR CLEAN-NOIV and MR CLEAN-MED investigators

Affiliations:

1. Department of Neurology, Erasmus MC University Medical Center, Rotterdam, The Netherlands
2. Department of Radiology & Nuclear Medicine, Erasmus MC University Medical Center, Rotterdam, The Netherlands
3. Department of Neurology, Amsterdam University Medical Centers, location AMC, Amsterdam, The Netherlands
4. Department of Radiology & Nuclear Medicine, Amsterdam University Medical Center, University of Amsterdam, Amsterdam, The Netherlands
5. Department of Biomedical Engineering & Physics, Amsterdam University Medical Center, University of Amsterdam, Amsterdam, The Netherlands
6. Department of Neurology, Maastricht University Medical Center, Cardiovascular Research Institute Maastricht (CARIM), Maastricht, The Netherlands
7. Department of Public Health, Erasmus MC University Medical Center, Rotterdam, The Netherlands

Table of Contents

Supplementary Figure

Figure I Flowchart of included patients………………………………………………… 3

Figure I mRS distribution any ICH vs. no ICH….…………………………………… 3

Supplementary Tables……………………………………………………………………… 4

Table I Classification of ICH based on Heidelberg Bleeding Criteria, stratified for asymptomatic ICH vs. symptomatic ICH……………………………………………. 4

Table I Subclassifications 'Other isolated hemorrhage (SDH/IVH/rPH)'…………… 4

Table II Subclassifications 'Combined HI with hemorrhage outside infarcted brain tissue'…………………….……………………………………………………….... 4

Table III Subclassifications 'Combined PH with hemorrhage outside infarcted brain tissue'…………………….……………………………………………………….. 5

Table IV Subclassifications 'Combined hemorrhages outside infarcted brain tissue'. 5

Supplementary Investigator List………………………………………………………… 6

MR CLEAN-NOIV Investigators……………..…………………………………….. 6

MR CLEAN-MED Investigators………..…………………………………………… 6

CONTRAST Investigators………………………………………………………… 7

List of affiliations…………………………………………………………………….. 8

van der Steen W, *et al. J NeuroIntervent Surg* 2023; 15:971–977. doi: 10.1136/jnis-2022-019474

Supplementary Figure

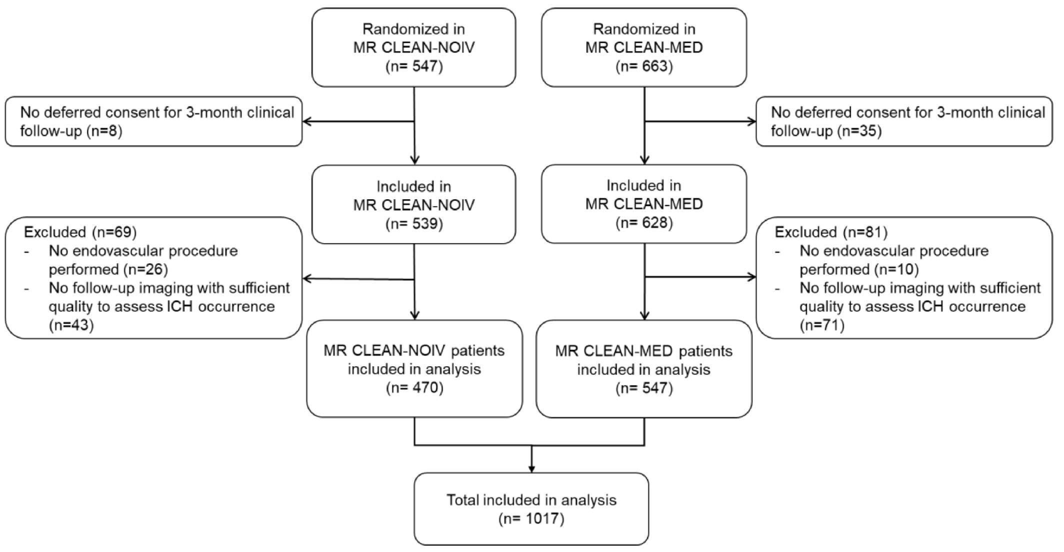

Figure I. Flowchart of included patients

ICH indicates intracranial hemorrhage

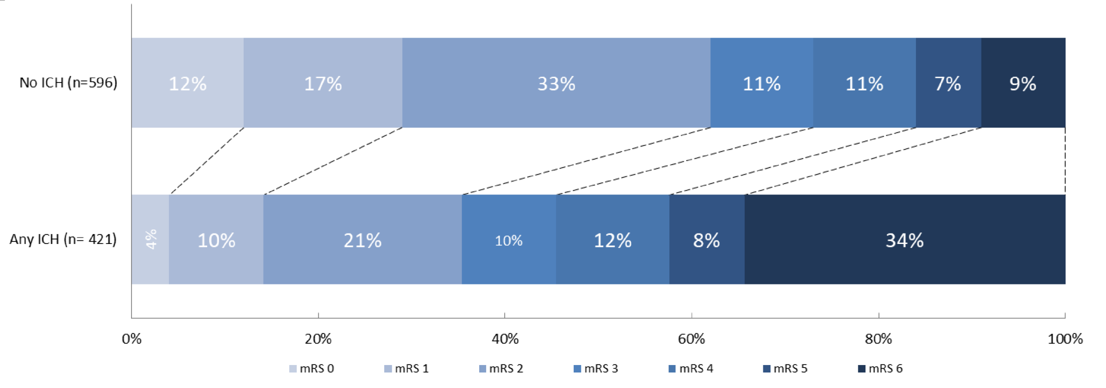

Figure II. Distribution of modified Rankin Scale (mRS) scores at 90 days for patients with no intracranial hemorrhage (ICH), and any ICH.

There was a significant shift towards worse functional outcomes for patients with any ICH versus patients with no ICH (adjusted common odds ratio (acOR), 0.59; 95% CI 0.46 to 0.76).

van der Steen W, *et al. J NeuroIntervent Surg* 2023; 15:971–977. doi: 10.1136/jnis-2022-019474

Supplementary Tables

Table I. Classification of intracranial hemorrhages (ICH) based on the Heidelberg Bleeding Classification, stratified for asymptomatic ICH vs. symptomatic ICH

	Asymptomatic ICH (n= 331)	Symptomatic ICH (n= 90)
Isolated hemorrhagic infarction type 1	99 (30%)	0 (0.0%)
Isolated hemorrhagic infarction type 2	74 (22%)	0 (0.0%)
Isolated parenchymal hematoma type 1	29 (8.8%)	3 (3.3%)
Isolated parenchymal hematoma type 2	12 (3.6%)	11 (12%)
Isolated subarachnoid hemorrhage	41 (12%)	3 (3.3%)
Other isolated hemorrhage (SDH/IVH/rPH)	10 (3.0%)	5 (5.6%)
Combined hemorrhagic infarction with hemorrhage outside infarcted brain tissue	32 (9.7%)	6 (6.7%)
Combined parenchymal hematoma with hemorrhage outside infarcted brain tissue	32 (9.7%)	59 (66%)
Combined hemorrhages outside infarcted brain tissue	2 (0.6%)	3 (3.3%)

Data are presented as n (%).

SDH indicates subdural hematoma; IVH, intraventricular hemorrhage; and rPH, remote parenchymal hematoma.

Table II. Subclassification of hemorrhages classified as ‘Other isolated hemorrhage (SDH/IVH/rPH)’

	Subdural hematoma	Intraventricular hemorrhage	Remote parenchymal hemorrhage
Other isolated hemorrhage (SDH/IVH/rPH)	2	3	10

Number of hemorrhages per subclassification are presented as N.

Table III. Subclassification of hemorrhages classified as ‘Combined hemorrhagic infarction with hemorrhage outside infarcted brain tissue’

	Subarachnoid hemorrhage	Subdural hematoma	Intraventricular hemorrhage	Remote parenchymal hemorrhage	Combined hemorrhages outside infarcted brain tissue
Hemorrhagic infarction type 1	9	2	0	4	3
Hemorrhagic infarction type 2	11	5	1	1	2

Number of hemorrhages per combined subclassification are presented as N.

van der Steen W, *et al. J NeuroIntervent Surg* 2023; 15:971–977. doi: 10.1136/jnis-2022-019474

Table IV. Subclassification of hemorrhages classified as 'Combined parenchymal hematoma with hemorrhage outside infarcted brain tissue'

	Subarachnoid hemorrhage	Subdural hematoma	Intraventricular hemorrhage	Remote parenchymal hemorrhage	Combined hemorrhages outside infarcted brain tissue
Parenchymal hematoma type 1	13	0	4	1	6
Parenchymal hematoma type 2	19	2	14	4	28

Number of hemorrhages per combined subclassification are presented as N.

Table V. Subclassification of hemorrhages classified as 'Combined hemorrhages outside infarcted brain tissue'

	Subdural hematoma	Intraventricular hemorrhage	Remote parenchymal hemorrhage
Subarachnoid hemorrhage	0	2	2
Subdural hematoma	X	0	0
Intraventricular hemorrhage	X	X	1

Number of hemorrhages per combined subclassification are presented as N. Combinations are only presented once. Cells with a combination that is already given in another cell are presented as 'X'.

van der Steen W, *et al. J NeuroIntervent Surg* 2023; 15:971–977. doi: 10.1136/jnis-2022-019474

Supplementary Investigator List

Investigators

MR CLEAN-NO IV Investigators

Principal investigators
Yvo Roos (MD, PhD),[1] Charles Majoie (MD, PhD)[1]

Study coordinators
Kilian Treurniet (MD),[1] Jonathan Coutinho (MD, PhD),[1] Bart Emmer (MD, PhD),[1] Natalie LeCouffe (MD),[1] Manon Kappelhof (MD),[1] Leon Rinkel (MD),[1] Agnetha Bruggeman (MD),[1]

Local principal investigators
Bob Roozenbeek (MD, PhD),[2] Adriaan van Es (MD, PhD),[2,26] Inger de Ridder (MD, PhD),[4] Wim van Zwam (MD, PhD),[4] Bart van der Worp (MD, PhD),[5] Rob Lo (MD, PhD),[5] Koos Keizer (MD, PhD),[6] Rob Gons (MD, PhD),[6] Lonneke Yo (MD, PhD),[6] Jelis Boiten (MD, PhD),[7] Ido van den Wijngaard (MD, PhD),[7] Geert Lycklama à Nijeholt (MD, PhD),[7] Jeannette Hofmeijer (MD, PhD),[8] Jasper Martens (MD),[8] Wouter Schonewille (MD, PhD),[9] Jan Albert Vos, (MD, PhD),[9] Anil Tuladhar (MD, PhD),[10] Floris Schreuder (MD, PhD),[10] Jeroen Boogaarts (MD, PhD),[10] Sjoerd Jenniskens (MD),[10] Karlijn de Laat (MD, PhD),[11] Lukas van Dijk (MD, PhD),[11] Heleen den Hertog (MD, PhD),[12] Boudewijn van Hasselt (MD),[12] Paul Brouwers (MD, PhD),[13] Emiel Sturm (MD),[13] Tomas Bulut (MD),[13] Michel Remmers (MD),[14] Anouk van Norden (MD),[14] Thijs de Jong (MD),[14] Anouk Rozeman (MD),[15] Otto Elgersma (MD, PhD),[15] Maarten Uyttenboogaart (MD, PhD),[16] Reinoud Bokkers (MD, PhD),[16] Julia van Tuijl (MD, PhD),[17] Issam Boukrab (MD),[17] Hans Kortman (MD),[17] Vincent Costalat (MD, PhD),[18] Caroline Arquizan (MD),[18] Robin Lemmens (MD, PhD),[19] Jelle Demeestere (MD),[20] Philippe Desfontaines (MD),[20] Denis Brisbois (MD),[20] Frédéric Clarençon (MD, PhD),[21] Yves Samson (MD)[21]

Executive and writing committee
Yvo Roos (MD, PhD),[1] Charles Majoie (MD, PhD),[1] Adriaan van Es (MD, PhD),[2,26] Diederik Dippel (MD, PhD),[2] Aad van der Lugt (MD, PhD),[2] Wim van Zwam (MD, PhD),[4] Jelis Boiten (MD, PhD),[7] Geert Lycklama à Nijeholt (MD, PhD),[7] Lonneke Yo (MD, PhD),[6] Koos Keizer (MD, PhD),[6] Jonathan Coutinho (MD, PhD),[1] Bart Emmer (MD, PhD),[1] Kilian Treurniet (MD),[1] Natalie LeCouffe (MD),[1] Manon Kappelhof (MD),[1]

Local MR CLEAN-NO IV collaborators

Data Safety Monitoring Board
Martin Brown (MD) – Chair,[22] Phil White (MD, PhD),[23] John Gregson (MD, PhD)[24]

Independent trial statistician
Daan Nieboer (MSc)[2]

MR CLEAN-MED investigators

Principal investigators
Diederik Dippel (MD, PhD),[2] Aad van der Lugt (MD, PhD)[2]

Study coordinators
Wouter van der Steen (MD),[2] Rob van de Graaf (MD, PhD),[2] Bob Roozenbeek (MD, PhD)[2]

Local principal investigators
Bob Roozenbeek (MD, PhD),[2] Adriaan van Es (MD, PhD),[2,26] Pieter Jan van Doormaal (MD),[2] Jonathan M. Coutinho (MD, PhD),[1] Bart Emmer (MD, PhD),[1] Inger de Ridder (MD, PhD),[4] Wim van Zwam (MD, PhD),[4] Bart van der Worp (MD, PhD),[5] Irene van der Schaaf (MD, PhD),[5] Rob Gons (MD, PhD),[6] Lonneke Yo (MD),[6] Jelis Boiten (MD, PhD),[7] Ido van den Wijngaard (MD, PhD),[7,26] Jeanette Hofmeijer (MD, PhD),[8] Jasper Martens (MD),[8] Wouter Schonewille (MD, PhD),[9] Jan Albert Vos, (MD, PhD),[9] Anil Man Tuladhar (MD, PhD),[10] Sjoerd Jenniskens (MD),[10] Karlijn de Laat (MD, PhD),[11] Lukas van Dijk (MD, PhD),[11] Heleen den Hertog (MD, PhD),[12] Boudewijn van Hasselt (MD),[12] Michel Remmers (MD),[14] Douwe Vos,[14] Anouk Rozeman (MD, PhD),[15] Otto Elgersma (MD,

PhD),[15] Maarten Uyttenboogaart (MD, PhD),[16] Reinoud Bokkers (MD, PhD),[16] Julia van Tuijl (MD, PhD),[17] Issam Boukrab (MD),[17] Benjamin Gory (MD, PhD),[27] Arturo Consoli (MD),[28] Mikaël Mazighi (MD, PhD),[29] Frederic Clarencon (MD, PhD),[30] Gaultier Marnat (MD),[21]

Executive and writing committee
Diederik Dippel (MD, PhD),[2] Aad van der Lugt (MD, PhD),[2] Rob van de Graaf (MD, PhD),[2] Wouter van der Steen (MD),[2] Bob Roozenbeek (MD, PhD),[2] Adriaan van Es (MD, PhD),[2] Yvo Roos (MD, PhD),[1] Charles Majoie (MD, PhD),[1] Robert van Oostenbrugge (MD, PhD),[4] Wim van Zwam (MD, PhD),[4] Julie Staals (MD, PhD),[4] Sjoerd Jenniskens (MD),[10] Lukas van Dijk (MD, PhD),[11] Heleen den Hertog (MD, PhD),[12]

Local MR CLEAN-MED collaborators

Data Safety Monitoring Board
Peter Rothwell (MD, PhD) – *Chair,*[31] Andrew Molyneux (MD, PhD),[31] Joanna Moschandreas (MD, PhD)[31]

Independent trial statistician
Daan Nieboer (MSc)[2]

Advisory Board
Gregory del Zoppo (MD, PhD)[32]

CONTRAST clinical trial collaborators

Research leaders
Diederik Dippel (MD, PhD),[2] Charles Majoie (MD, PhD)[1]

Consortium coordinator:
Rick van Nuland, (PhD)[3]

Imaging assessment committee
Charles Majoie (MD, PhD) – *Chair*,[1] Aad van der Lugt (MD, PhD) – *Chair*,[2] Adriaan van Es, (MD, PhD),[2,26] Pieter-Jan van Doormaal (MD),[2] René van den Berg, (MD, PhD),[1] Ludo Beenen (MD, PhD),[1] Bart Emmer (MD, PhD),[1] Stefan Roosendaal (MD, PhD),[1] Wim van Zwam (MD, PhD),[4] Alida Annechien Postma (MD, PhD),[33] Lonneke Yo (MD, PhD),[6] Menno Krietemeijer (MD),[6] Geert Lycklama (MD, PhD),[7] Jasper Martens (MD),[8] Sebastiaan Hammer (MD, PhD),[10] Anton Meijer (MD, PhD),[10] Reinoud Bokkers (MD, PhD),[16] Anouk van der Hoorn (MD, PhD),[16] Ido van den Wijngaard (MD, PhD),[7,26] Albert Yoo (MD, PhD),[25] Dick Gerrits (MD)[13]

Adverse event committee
Robert van Oostenbrugge (MD, PhD) – *Chair*,[4] Bart Emmer (MD, PhD),[1] Jonathan M. Coutinho (MD, PhD),[1] Martine Truijman (MD, PhD),[4] Julie Staals (MD, PHD),[4] Bart van der Worp (MD, PhD),[5] J. Boogaarts (MD, PhD),[10] Ben Jansen (MD, PhD),[17] Sanne Zinkstok (MD, PhD)[34]

Outcome assessment committee
Yvo Roos (MD, PhD) – *Chair*,[1] Peter Koudstaal (MD, PhD),[2] Diederik Dippel (MD, PhD),[2] Jonathan M. Coutinho (MD, PhD),[1] Koos Keizer (MD, PhD),[5] Sanne Manschot (MD, PhD),[7] Jelis Boiten (MD, PhD),[7] Henk Kerkhoff (MD, PhD),[15] Ido van den Wijngaard (MD, PhD)[7,26]

Data management group
Hester Lingsma (PhD),[2] Diederik Dippel (MD, PhD),[2] Vicky Chalos (MD),[2] Olvert Berkhemer (MD, PhD)[1,2]

Imaging data management
Aad van der Lugt (MD, PhD),[2] Charles Majoie (MD, PhD),[1] Adriaan Versteeg,[2] Lennard Wolff (MD),[2] Matthijs van der Sluijs (MD),[2] Henk van Voorst (MD),[1] Manon Tolhuisen (MSc),[1]

Biomaterials and translational group
Hugo ten Cate (MD, PhD),[4] Moniek de Maat (PhD),[2] Samantha Donse-Donkel (MD),[2] Heleen van Beusekom (PhD),[2] Aladdin Taha (MD),[2] Aarazo Barakzie (MD)[2]

Local collaborators
Vicky Chalos (MD, PhD),[2] Rob van de Graaf (MD, PhD),[2] Wouter van der Steen (MD),[2] Aladdin Taha (MD),[2] Samantha Donse-Donkel (MD),[2] Lennard Wolff (MD),[2] Kilian Treurniet (MD),[1] Sophie van den Berg (MD),[1] Natalie LeCouffe (MD),[1] Manon Kappelhof (MD),[1] Rik Reinink (MD),[1] Manon Tolhuisen (MD),[1] Leon Rinkel (MD),[1] Josje Brouwer (MD),[1] Agnetha Bruggeman (MD),[1] Henk van Voorst (MD),[1] Robert-Jan Goldhoorn (MD),[4] Wouter Hinsenveld (MD),[4] Anne Pirson (MD),[4] Susan Olthuis (MD),[4] Simone Uniken Venema (MD),[5] Sjan Teeselink (MD),[10] Lotte Sondag (MD),[10] Sabine Collette (MD)[16]

Research nurses
Martin Sterrenberg,[2] Naziha El Ghannouti,[2] Laurine van der Steen,[1] Sabrina Verheesen,[4] Jeannique Vranken,[4] Ayla van Ahee,[5] Hester Bongenaar,[6] Maylee Smallegange,[6] Lida Tilet,[6] Joke de Meris,[7] Michelle Simons,[8] Wilma Pellikaan,[9] Wilma van Wijngaarden,[9] Kitty Blauwendraat,[9] Yvonne Drabbe,[11] Michelle Sandiman-Lefeber,[11] Anke Katthöfer,[11] Eva Ponjee,[12] Rieke Eilander,[12] Anja van Loon,[14] Karin Kraus,[14] Suze Kooij,[15] Annemarie Slotboom,[15] Marieke de Jong,[16] Friedus van der Minne,[16] Esther Santegoets[17]

Study monitors
Leontien Heiligers[2], Yvonne Martens,[2] Naziha El Ghannouti[2]

Affiliations

[1] Amsterdam UMC, University of Amsterdam, Amsterdam, the Netherlands;
[2] Erasmus MC University Medical Center, Rotterdam, the Netherlands;
[3] Lygature, Utrecht, the Netherlands;
[4] Cardiovascular Research Institute Maastricht (CARIM), Maastricht University Medical Center, Maastricht, The Netherlands;
[5] University Medical Center Utrecht, Brain Center, Utrecht, the Netherlands;
[6] Catharina Hospital, Eindhoven, the Netherlands;
[7] Haaglanden Medical Center, the Hague, the Netherlands;
[8] Rijnstate Hospital, Arnhem, the Netherlands;
[9] St. Antonius Hospital, Nieuwegein, the Netherlands;
[10] Radboud University Medical Center, Nijmegen, the Netherlands;
[11] HagaZiekenhuis, the Hague, the Netherlands;
[12] Isala Klinieken, Zwolle, the Netherlands;
[13] Medisch Spectrum Twente, Enschede, the Netherlands;
[14] Amphia Hospital, Breda, the Netherlands;
[15] Albert Schweitzer Hospital, Dordrecht, the Netherlands;
[16] University Medical Center Groningen, the Netherlands;
[17] Elisabeth-TweeSteden Hospital, Tilburg, the Netherlands;
[18] Centre Hospitalier Universitaire de Montpellier, Montpellier, France;
[19] University Hospitals Leuven, Leuven, Belgium;
[20] Centre Hospitalier Chrétien, Liège, Belgium;
[21] Pitié-Salpêtrière hospital APHP-Sorbonne Université Paris France, Paris, France;
[22] National Hospital for Neurology and Neurosurgery, London, United Kingdom;
[23] Institute of Neuroscience and Newcastle University Institute for Ageing, Newcastle University, Newcastle, UK;
[24] London School of Hygiene & Tropical Medicine, London, United Kingdom;
[25] Texas Stroke Institute, Plano, Texas, United States of America
[26] Leiden Universitair Medisch Centrum, Leiden, the Netherlands
[27] University Hospital of Nancy, Nancy, France
[28] Foch Hospital, Suresnes, France
[29] Fondation Rothschild Hospital, Paris, France
[30] University Hospital of Bordeaux, Bordeaux, France
[31] John Radcliffe Hospital, Oxford, United Kingdom
[32] University of Washington, Seattle, Washington, United States
[33] School for Mental Health and Sciences (Mhens), Maastricht University Medical Center, Maastricht, The Netherlands
[34] TerGooi, Hilversum, The Netherlands
